# New Trends in Dermoscopy to Minimize the Risk of Missing Melanoma

**DOI:** 10.1155/2012/820474

**Published:** 2012-10-08

**Authors:** Aimilios Lallas, Zoe Apalla, Georgios Chaidemenos

**Affiliations:** ^1^State Clinic of Dermatology, Hospital of Skin and Venereal Diseases, 124 Delfon Street, 54643 Thessaloniki, Greece; ^2^Private Practice, 8 Kosma Aitolou Street, 54643 Thessaloniki, Greece

## Abstract

During the last decades, induction of dermoscopy in the clinical setting resulted in significant modifications in the management of melanocytic lesions. Indeed, the dermatoscope reveals a fascinating world of morphologic structures invisible to the naked eye, adding valuable information to a clinician evaluating a mole. However, since the technique counts only a couple of decades, new research data are continuously gathering and modify the “optimal” management of melanocytic lesions. In the present paper, we summarize the latest trends in dermoscopy concerning early melanoma diagnosis, management of nodular lesions, diagnosis of mucosal melanoma, and digital followup.

## 1. Introduction

During the last decades, induction of dermoscopy in the clinical setting resulted in significant modifications in the management of melanocytic lesions [1]. Incidence of melanoma increases more rapidly than any other cancer, and this has partially been attributed to the development of highly sensitive diagnostic techniques—mainly dermoscopy—, which allowed the detection of clinically not identifiable tumors [2]. Indeed, the dermatoscope reveals a fascinating world of morphologic structures invisible to the naked eye, adding valuable information to a clinician evaluating a mole.

The controversy whether dermoscopy improves diagnostic accuracy when assessing melanocytic lesions belongs to the history, since several lines of evidence clearly demonstrate that use of dermoscopy both enhances melanoma detection and decreases the number of unnecessary excisions [3]. Additionally to its use for diagnostic purposes, dermoscopy, by revealing unknown information on morphology, enhanced the understanding of nevogenesis and generated new concepts and theories [4]. However, since the technique counts only a couple of decades, new research data are continuously gathering and modify the “optimal” management of melanocytic lesions.

The aim of this paper was to summarize the latest trends in dermoscopy for minimizing the risk to miss melanoma. 

## 2. New Trends in Early Melanoma Detection

With the introduction of dermoscopy, the new morphologic world of features and structures had to be explored. In order to enhance the utility of the method in the clinical setting, various diagnostic models and algorithms have been developed [5–7]. Indeed, application of these algorithms resulted in significant improvement of the diagnostic accuracy of clinicians evaluating melanocytic lesions [8–10]. Simplified diagnostic approaches were also proposed, aiming to facilitate the use of dermoscopy from less experienced clinicians and nondermatologists and thus widen the application field of the method [11]. Additionally to their applicability in daily routine, algorithms are also considered of value for educational purposes, providing a mathematic and comprehensive model and guiding the inexperienced clinician on how to deal with the various dermoscopic features. 

Several lines of evidence, including meta-analyses and randomized trials, have validated the value of dermoscopy in improving the accuracy of melanoma detection [12–16]. Recently, a 10-year multicenter survey confirmed that, in specialized clinical settings, the use of dermoscopy results in reduction of excised nevi and increased number of diagnosed melanomas [17]. 

Despite the high level of existing evidence concerning the value of dermoscopy in the management of melanocytic lesions, limitations and drawbacks related to the method do exist. Haenssle et al. measured the performance of the seven-point checklist during 10 years of prospective surveillance of patients at increased melanoma risk [18]. They assessed the method as highly specific but less sensitive, since 38% of melanomas did not reach the threshold of 3 points. Skvara et al. evaluated retrospectively the baseline images of nevi and early melanomas that were finally excised because of changes over time [19]. They concluded that none of the common dermoscopic algorithms could reliably differentiate between the two entities and suggested that dermoscopy is of relatively limited value in the diagnosis of very early melanomas. 

A possible explanation is that the dermoscopic algorithms were constructed more than a decade ago, based on clear-cut melanomas and clinically equivocal nevi. In other words, they were tested on lesions that were already worrisome from a clinical point of view. Today, dermoscopy is not considered a second-line method for further evaluation of clinically equivocal lesions, but, instead, it represents a first-line tool for screening of patients with few or multiple nevi [1]. Under this scenario, dermoscopy is often employed to detect early melanoma among a plethora of clinically banal moles. Undoubtedly, dermoscopic criteria of melanoma appear earlier than the clinical characteristics, and, subsequently, earlier diagnosis via dermoscopy is feasible when the tumor is still clinically indolent. However, in very early stages, when the morphologic structures of melanoma have not yet fully developed, they may be overlooked even dermoscopically. 

This prompted Argenziano et al. to revise the seven-point checklist and lower the threshold for excision from 3 points to 1, achieving higher rates of sensitivity of melanoma detection, with a consequent loss of specificity [20]. In the daily clinical setting, the authors recommend using the revised dermoscopic algorithm in conjunction with clinical information and followup, in order to minimize the risk to miss a melanoma. The latter recommendation reflects today's trend in melanoma diagnostics, namely to focus all efforts towards the earliest possible detection of the cancer.

## 3. New Trends in Dermoscopic Diagnosis of Nodular Melanoma (NM)

NM is an aggressive, potentially lethal tumor and represents the most important threat in melanoma diagnostics, since if it is overlooked and left untreated, it might result in severe consequences [21]. Clinically, NM usually lacks the classic ABCD criteria (asymmetry, border, color, and diameter) and might be difficult to differentiate from benign tumors, such as vascular tumors, dermal and blue nevi, dermatofibromas, or even seborrheic keratoses [22]. EFG criteria (elevation, firmness, and growth) have been suggested to enhance the clinical diagnosis of NM, but their diagnostic accuracy remains currently unknown [22]. 

Dermoscopy has been shown to enable detection of ABCD-negative melanomas, but most dermoscopic features have been described in the context of superficial spreading melanoma [23]. In addition, several well-known dermoscopic criteria of melanoma cannot be detected in purely nodular tumors, since they histopathologically correspond to melanin in the epidermis or at the dermoepidermal junction. 

Aiming to address this problem, Argenziano et al. described a new predictor of nodular melanoma, namely, the presence of blue and black color within the lesion [24]. Blue-black color is suggested to reflect the combination of pigment localized in the middeep dermis (blue) and the epidermis (black). The authors found a higher sensitivity for blue-black color compared to the standard melanoma criteria, while a combined method reached the highest accuracy for the diagnosis of NM (84.6% sensitivity and 80.5% specificity). 

Following the latter study, the blue-black feature should be added to the standard melanoma criteria when evaluating nodular lesions. However, since still approximately 15% of NMs cannot be detected even with the addition of the blue-black feature, the “if in doubt, cut it out” approach represents the safest strategy in the management of nodular lesions.

## 4. New Trends in Dermoscopic Diagnosis of Mucosal Melanoma

Evaluation of pigmented lesions located on the mucosa is considered problematic because of the high rates of benign disorders mimicking melanoma, the very low rates of the latter, and the lack of pathognomonic clinical features capable to distinguish between the two [25, 26]. While dermoscopy is widely used for the diagnosis of pigmented and nonpigmented lesions of the skin, until recently its applicability on pigmented mucosal lesions was not well established. The largest available studies, conducted first by Lin et al. [27] and second by Ronger-Savle et al. [28], had included 40 mucosal and 68 vulvae lesions, respectively. 

In order to obtain a larger number of cases, the International Dermoscopy Society (IDS) conducted a multicenter retrospective, observational study to better characterize the dermoscopic features of benign and malignant pigmented mucosal lesions [29]. Lesions from various anatomic sites, namely, lip, labia, glans, praeputium, and other anogenital areas, were included. Evaluators scored the dermoscopic patterns (dots, globules, clods, circles, lines, or structureless) and colors (brown, black, blue, gray, red, purple, and white), and correlated them with the histopathologic characteristics.

Based on their results, the authors concluded that the combination of blue, gray, or white color with structureless zones was the strongest indicator when differentiating melanoma from benign mucosal lesions by dermoscopy. Analyzing further the recorded data, they suggested that early signs of a mucosal melanoma could include the presence of structureless parts and gray color, while multiple patterns and additional colors, especially blue or white, may represent common additional late-stage signs in larger lesions. The age of the patient, as shown in the study, may also be of help in the differential diagnosis, since melanomas were more common in advanced ages (mean age of 60.1 years for mucosal melanomas versus 43.2 years for benign pigmented lesions).

Summarizing the latest data concerning mucosal melanoma, we can recommend increased awareness of physicians when blue, gray, or white color in the background of a structureless zone is recognized in dermoscopy.

## 5. New Trends in Digital Dermoscopy

Digital dermoscopy has been shown to enable the identification of early melanomas lacking specific dermoscopic criteria, by revealing morphologic alterations at sequential monitoring [30]. This is especially relevant in the background of patients with multiple nevi. It is well known that a significant proportion of these individuals' nevi will show some degree of atypia and accordingly may be unnecessarily excised, while newly arising melanoma may be missed among the plethora of their clinically ugly nevi. Under this scenario, it has been demonstrated that early recognition of melanoma is highly enhanced by followup with digital dermoscopy, which allows observation of dermoscopic changes over time [30, 31]. In the study by Skvara et al. [19], 89% (56/63) of melanomas detected by digital monitoring showed no evidence of melanoma when standard dermoscopic criteria were used. 

Apart from improving early melanoma detection, digital dermoscopy is also of value in minimizing the frequency of excisions of benign lesions [32]. The latter may be partially explained by the fact that digital dermoscopy is a convenient tool for appliance of the comparative approach, which is already known to significantly reduce unnecessary excisions of clinically and dermoscopically worrisome nevi [33]. The comparative approach, which is also known as the concept of “signature nevus” or “the ugly duckling sign,” refers to the observation that the majority of a given individual's nevi exhibit a similar dermoscopic pattern, while melanoma will reveal different features. This knowledge aids a clinician to avoid excisions of nevi with equivocal dermoscopic morphology which would be removed if they were not compared to the predominant nevi pattern of the patient. A recent study showed that application of the comparative approach results in significant reduction of nevi excisions [32]. Similarly, Salerni et al. recently showed that using digital dermoscopic followup in patients at high risk of melanoma minimizes the ratio of unnecessarily excised lesions [34].

Although the value of sequential digital imaging is clear, defining the optimal followup intervals represents a conflicting issue. Menzies et al. [35] proposed in 2001 a short-term monitoring protocol, suggesting that morphologic evolution of melanoma can be recognized in three-month time, while only a small proportion of nevi exhibit morphologic changes in such a short period. Consequently, the authors concluded that detected morphologic changes at short followup require excision of the lesion. Altamura et al. [36] investigated whether 6 weeks could replace 3 months for sequential digital monitoring and concluded that 3 months remains the optimal interval, in terms of sensitivity of melanoma diagnosis. Haenssle et al. [37] proposed 3 months as the recommended followup period for patients with familial atypical mole and multiple melanoma (FAMMM) syndrome and 6–12 months for those with atypical mole syndrome. 

Argenziano et al. [38] recently described the existence of a slow-growing subgroup of melanomas which may be undetectable at short-term followup. The subtle morphologic changes occurring in the latter subgroup of melanomas may require long-term surveillance to be identified. The authors suggested that in the context of a patient with multiple atypical nevi, the first reexamination should be scheduled at 3 months after the baseline visit, while prolonged annual monitoring should be performed to avoid missing indolent, slow-growing melanomas.

## 6. Conclusion

Dermoscopy is nowadays considered an irreplaceable part of the clinical examination for detection of melanoma. Given that new data are continuously gathering and modify the optimal management of melanocytic lesions, clinicians should closely follow up the newest trends in order to minimize the risk of missing melanoma.
